# EZH2 Depletion Blocks the Proliferation of Colon Cancer Cells

**DOI:** 10.1371/journal.pone.0021651

**Published:** 2011-07-13

**Authors:** Bettina Fussbroich, Nina Wagener, Stephan Macher-Goeppinger, Axel Benner, Maria Fälth, Holger Sültmann, Angela Holzer, Karin Hoppe-Seyler, Felix Hoppe-Seyler

**Affiliations:** 1 Molecular Therapy of Virus-Associated Cancers, German Cancer Research Center (DKFZ), Heidelberg, Germany; 2 Department of Urology, University of Heidelberg, Heidelberg, Germany; 3 Institute of Pathology, University of Heidelberg, Heidelberg, Germany; 4 Division of Biostatistics, German Cancer Research Center, Heidelberg, Germany; 5 Cancer Genome Research, German Cancer Research Center, Heidelberg, Germany; Roswell Park Cancer Institute, United States of America

## Abstract

The Enhancer of Zeste 2 (EZH2) protein has been reported to stimulate cell growth in some cancers and is therefore considered to represent an interesting new target for therapeutic intervention. Here, we investigated a possible role of EZH2 for the growth control of colon cancer cells. RNA interference (RNAi)-mediated intracellular EZH2 depletion led to cell cycle arrest of colon carcinoma cells at the G1/S transition. This was associated with a reduction of cell numbers upon transient transfection of synthetic *EZH2*-targeting siRNAs and with inhibition of their colony formation capacity upon stable expression of vector-borne siRNAs. We furthermore tested whether EZH2 may repress the growth-inhibitory *p27* gene, as reported for pancreatic cancer. However, expression analyses of colon cancer cell lines and colon cancer biopsies did not reveal a consistent correlation between EZH2 and p27 levels. Moreover, EZH2 depletion did not re-induce *p27* expression in colon cancer cells, indicating that *p27* repression by EZH2 may be cell- or tissue-specific. Whole genome transcriptome analyses identified cellular genes affected by EZH2 depletion in colon cancer cell lines. They included several cancer-associated genes linked to cellular proliferation or invasion, such as *Dag1*, *MageD1*, *SDC1*, *Timp2*, and *Tob1*. In conclusion, our results demonstrate that EZH2 depletion blocks the growth of colon cancer cells. These findings might provide benefits for the treatment of colon cancer.

## Introduction

The Enhancer of Zeste Homolog 2 (EZH2) protein is a core component of the Polycomb Repressive Complex2 (PRC2) and modifies transcription at the epigenetic level by affecting histone and DNA methylation [1]. EZH2 is overexpressed in several malignancies, including major human cancers, such as prostate cancer, breast cancer, pancreatic cancer, renal cell carcinoma, or cervical cancer [2]–[6].

There is experimental evidence that *EZH2* can directly contribute to carcinogenesis by acting as a *bona fide* oncogene. Specifically, for certain cancer entities, it has been reported that EZH2 stimulates cell proliferation, blocks apoptosis, promotes cell invasion and metastasis, activates tumor angiogenesis, and induces tumors in mouse models [2]–[11]. These findings suggest that EZH2 inhibition may represent an attractive novel strategy for epigenetic cancer therapy [1], [12].

More recently, however, there is also data suggesting that EZH2 could act as a tumor suppressor protein in certain tissues [13]. Homozygous inactivating *EZH2* mutations were detected in a portion of myeloid malignancies [14], [15], raising the possibility that EZH2 may either exert pro- or anti-oncogenic activities, in a cell type-dependent manner [16]. Another level of complexity is added by the detection of heterozygous *EZH2* mutations in a portion of lymphomas of germinal-center origin [13]. In this case, the mutant protein appears to increase the level of H3K27 methylation, a critical downstream target of EZH2, by acting in conjunction with the wild-type protein expressed from the unmutated allele [17].

Colorectal cancer is the fourth most common cancer form in humans. Each year, more than 1,200,000 individuals will develop the disease and over 600,000 will die from it [18]. Despite the high biomedical significance of this tumor, investigations of the EZH2 status and function in colon cancer cells are sparse and partly contradictory. For example, whereas EZH2 was consistently reported to be overexpressed in colon cancers, EZH2 expression levels correlated positively [19], negatively [20], [21], or not at all [22], with the survival of colon cancer patients. Moreover, to our knowledge, only one functional study investigated the role of EZH2 for the growth of colon cancer cells, but failed to see an effect upon *EZH2* gene silencing [22]. This finding is in strong contrast to the growth-promoting role of EZH2 reported for several other cancer entities [2]–[6]. In the present work, we addressed this issue by analyzing EZH2 expression in colon cancer cells *in vitro* and *in vivo*, and by investigating the contribution of EZH2 to the growth of colorectal cancer cell lines.

## Results

### Expression of EZH2 in colon cancer cells *in vitro* and RNA interference-mediated *EZH2* repression

**Figure 1:**
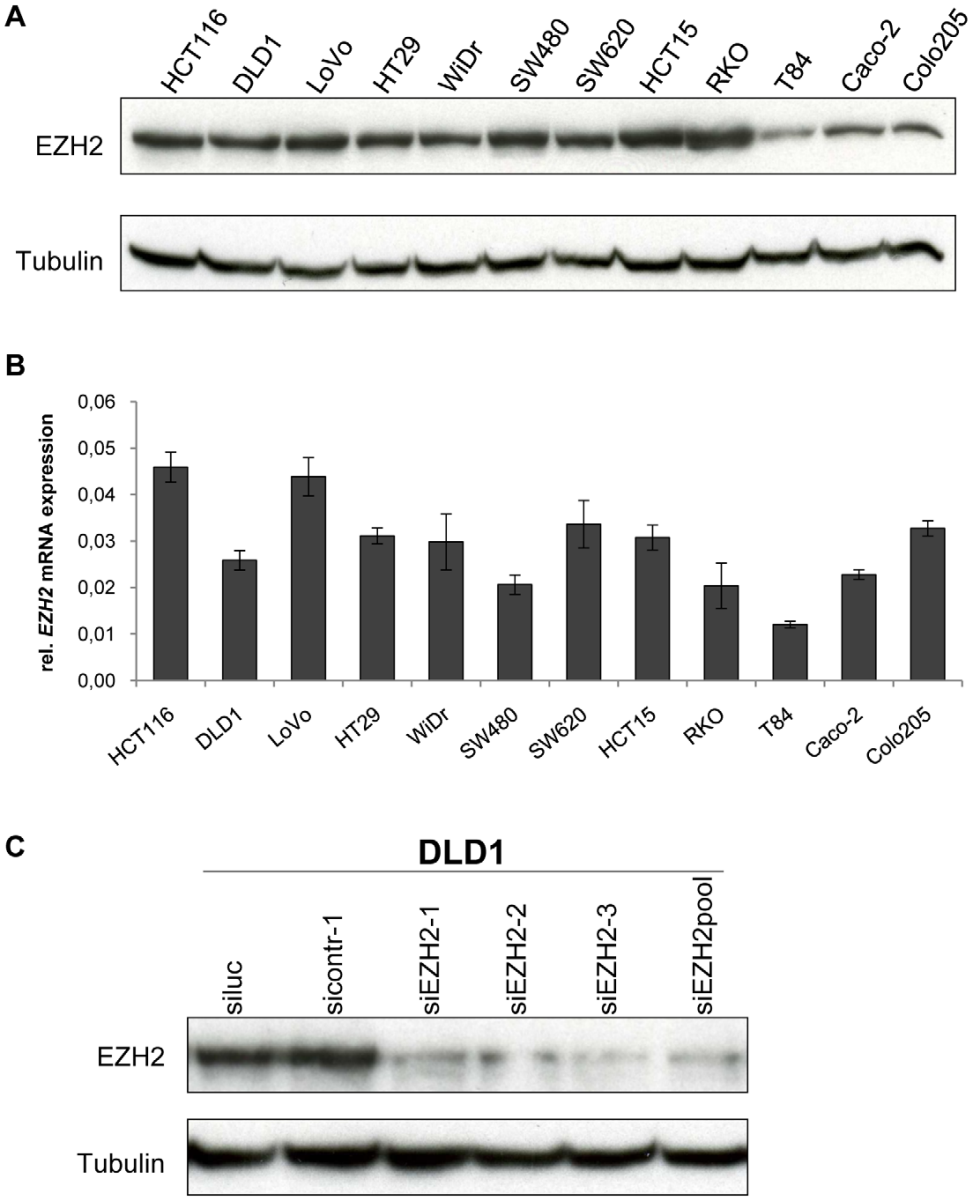
EZH2 expression in colon cancer cell lines. **A** Immunoblot analysis of EZH2 protein expression. Tubulin, loading control. **B** qRT-PCR analyses of *EZH2* mRNA expression. Data are presented as fold differences in gene expression, normalized to a housekeeping gene index. Standard deviations from two reverse transcription replicates are indicated. **C** Modulation of EZH2 protein expression by RNAi. EZH2 expression was determined by immunoblot analysis 48 hours after transfection with *EZH2*-targeting siRNAs or control siRNAs, as indicated. siEZH2pool: pooled *EZH2*-targeting siRNAs. Tubulin, loading control.

In order to investigate the expression of EZH2 in colon cancer cells *in vitro*, we analyzed a panel of twelve tumor-derived colon cancer cell lines by immunoblotting and qRT-PCR. All tested cell lines expressed readily detectable amounts of EZH2 protein (Figure 1A) and *EZH2* mRNA (Figure 1B). For subsequent RNA interference (RNAi) analyses, we generated three synthetic siRNAs targeting different regions of the *EZH2* transcript. All three siRNAs efficiently blocked EZH2 expression (Figure 1C). Since potential off-target effects of individual siRNAs can be reduced by siRNA pooling [23], [24], we also tested a pool consisting of all three *EZH2*-targeting siRNAs. This siRNA pool also efficiently blocked EZH2 expression (Figure 1C) and was used for further functional analyses.

### *EZH2* repression results in G1 arrest and growth inhibition of colon cancer cells

**Figure 2:**
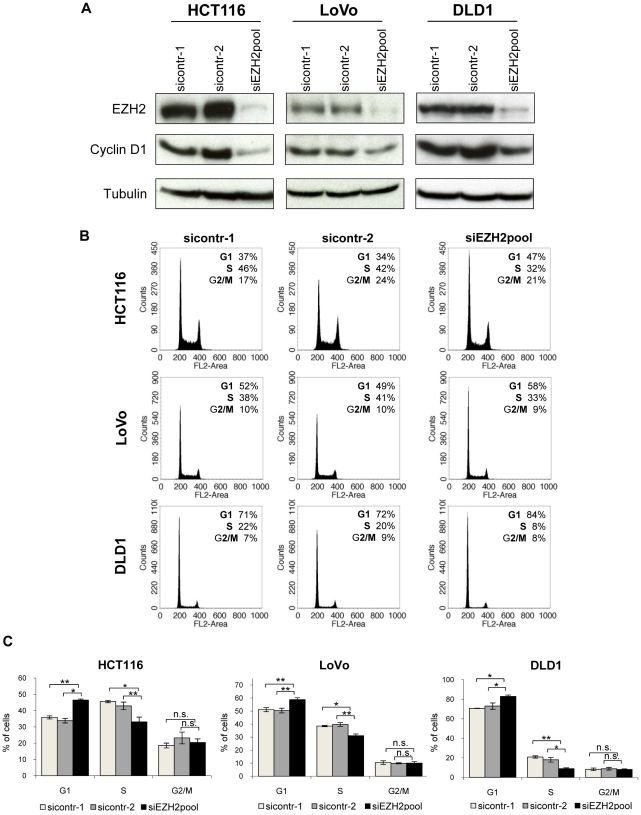
EZH2 depletion leads to cell cycle arrest of colon cancer cell lines. **A** Immunoblot analyses of HCT116, DLD1, and LoVo cells showing efficient downregulation of EZH2 expression by RNAi. Cyclin D1 levels are indicated. Tubulin, loading control. **B** Cell cycle analyses by FACS. Cells were treated with two control siRNAs or with *EZH2*-targeting siRNAs. Percentages of cells in the G1, S, or G2/M phases of the cell cycle are indicated. **C** Compilation of cell cycle analyses from three independent experiments. Standard deviations are indicated. Asterisks equal p≤0.05, double asterisks equal p≤0.01, n.s. not significant.

Next, we tested the effect of RNAi-mediated *EZH2* repression on the growth of HCT116, LoVo, and DLD1 colorectal cancer cells. siRNA-treatment resulted in a strong reduction of EZH2 levels in all tested colon carcinoma cell lines and, as previously reported for other cells [7], in a concomitant decrease of cyclin D1 expression (Figure 2A).

Cell cycle analyses by fluorescence activated cell sorting (FACS) were performed in parallel. They revealed a statistically significant increase in G1 populations and a concomitant decrease in S phase populations, upon *EZH2* repression. Typical FACS curves are shown in Figure 2B, a compilation of the results of three independent experiments is depicted in Figure 2C. These results indicate that *EZH2* repression induces cell cycle arrest at the G1/S boundary and therefore may act antiproliferative in colon cancer cells.

**Figure 3:**
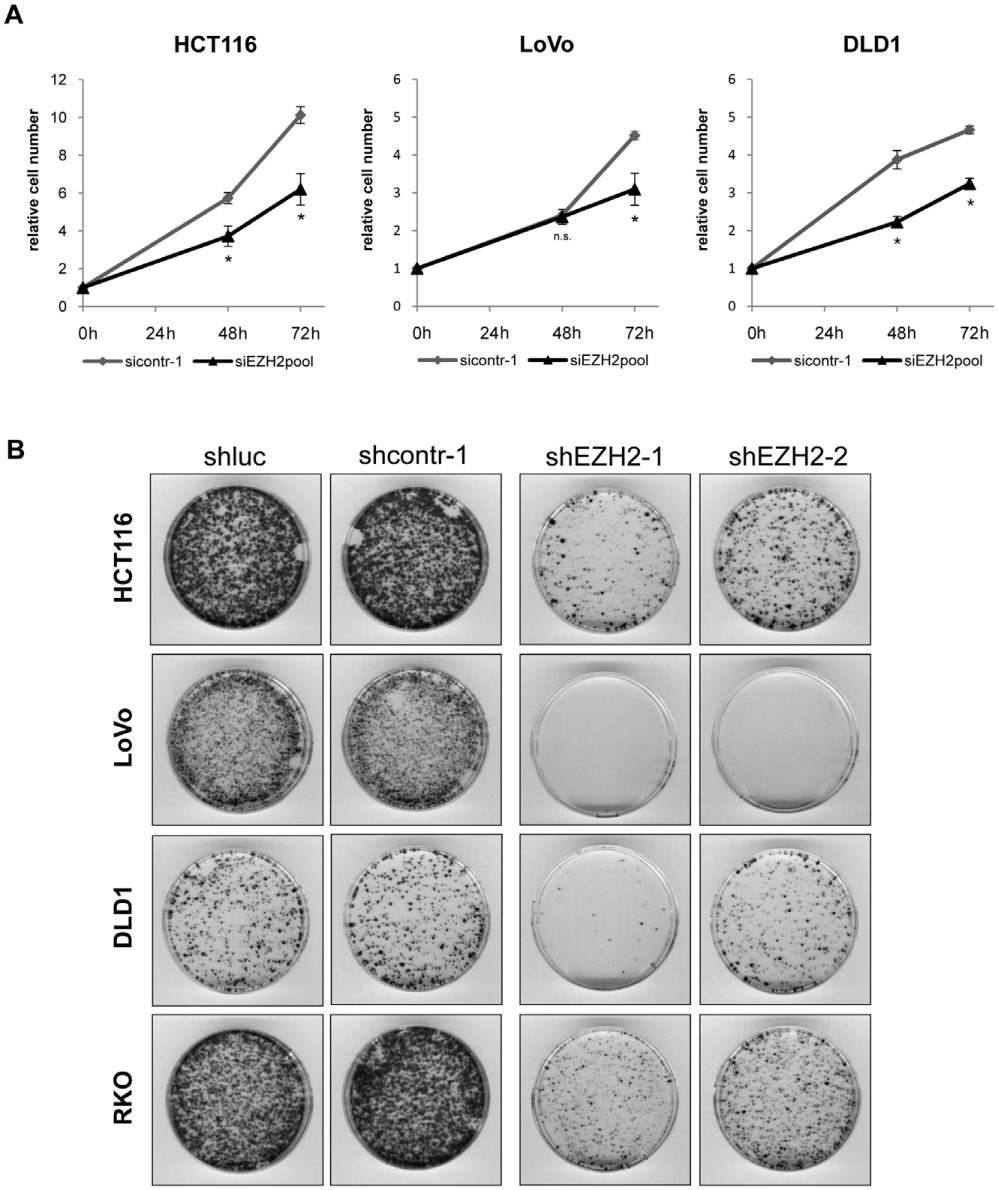
EZH2 depletion leads to growth inhibition of colon cancer cell lines. **A** Cell counts following transient transfection with synthetic control siRNA (sicontr-1) or *EZH2*-targeting siRNAs (siEZH2 pool). Graphs represent relative cell numbers, at the indicated time points after siRNA transfection. Cell numbers at transfection (time point 0) were set as 1.0. Experiments were performed in triplicates, standard deviations are indicated. Asterisks equal p≤0.05, n.s. not significant. **B** Colony formation assays. HCT116, LoVo, DLD1, and RKO cells were stably transfected with plasmids expressing two different shRNAs against *EZH2* (pCEP-shEZH2-1, pCEP-shEZH2-2) or two control shRNAs (pCEP-shluc or pCEP-shcontr-1). Experiments were independently repeated at least thrice, with consistent results.

To further address this issue, cell count analyses of colon cancer cell lines were performed. RNAi-mediated inhibition of *EZH2* expression led to a significant reduction of cell numbers, which was clearly visible 48–72 hours following transfection of synthetic siRNAs (Figure 3A), indicating that *EZH2* silencing results in growth inhibition of colon cancer cells.

To validate the antiproliferative effect of *EZH2* inhibition in colon cancer cells by an independent method, we performed colony formation assays. Two different *EZH2*-targeting siRNAs were stably expressed from selectable expression vectors in HCT116, LoVo, DLD1, and RKO cells, for 13 to 15 days. Both *EZH2*-targeting siRNAs led to a clear reduction of the colony formation capacity of all tested colon cancer cell lines versus control siRNA-treated cells (Figure 3B). Morphological aspects, FACS profiles, and terminal deoxynucleotidyl transferase dUTP nick end labeling (TUNEL) analyses did not provide evidence for increased apoptosis of colon cancer cells upon RNAi-mediated *EZH2* repression (data not shown).

Taken together, these results indicate that EZH2 depletion induces cell cycle arrest in the G1 phase and inhibits the growth of colon cancer cells.

### Tissue Micro Array analysis of EZH2 expression in colon adenomas and cancers

**Figure 4:**
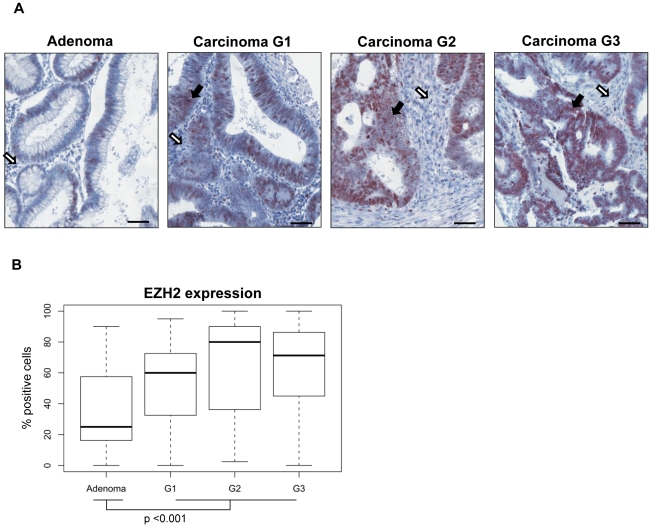
*In vivo* expression of EZH2 in colon carcinomas. **A** Immunohistochemical analyses of colon adenomas and colon cancer biopsies representing increasing degrees of histological dedifferentiation (G1–G3). Black arrows: carcinoma; white arrows: connective tissue. Scale bars, 50 µm. **B** Box plot of EZH2 protein expression. Expression levels of EZH2 were significantly increased in carcinomas when compared with adenomas (p<0.001). Differences for EZH2 expression between G1, G2, and G3 carcinomas were not significant (p = 0.185).

Previous studies have shown that EZH2 is significantly overexpressed in colon cancers when compared to normal colon tissue [19]–[22]. However, data comparing EZH2 expression in benign colon adenomas versus colon cancer is, to our knowledge, not yet available. We therefore performed immunohistochemical analyses employing a tissue microarray encompassing 24 adenomas, 25 G1 carcinomas, 24 G2 carcinomas, and 24 G3 carcinomas. In comparison to colon adenomas, EZH2 expression was significantly increased in colon carcinomas (Figs. 4A and 4B). Analyses of colon cancers representing different degrees of histological dedifferentiation (increasing from G1–G3) revealed a trend for a further increase of EZH2 expression for less differentiated cancers, which, however, was not statistically significant (Figure 4B).

### EZH2 and p27 expression do not correlate in colon cancer

The cyclin-dependent kinase inhibitor p27 (also named Kip1) is a growth inhibitory protein that blocks cell cycle progression at the G1/S transition [25]. In colon cancers, p27 levels are frequently low [26], [27]. Interestingly, it was recently reported that EZH2 depletion led to p27 re-expression in pancreatic cancer cells, indicating that EZH2 may contribute to tumor cell proliferation by repressing *p27* [4]. In view of our findings that EZH2 promotes cell proliferation and stimulates G1/S cell cycle progression of colon cancer cells, we addressed the question whether *p27* is repressed by EZH2 in colon cancer as well.

**Figure 5:**
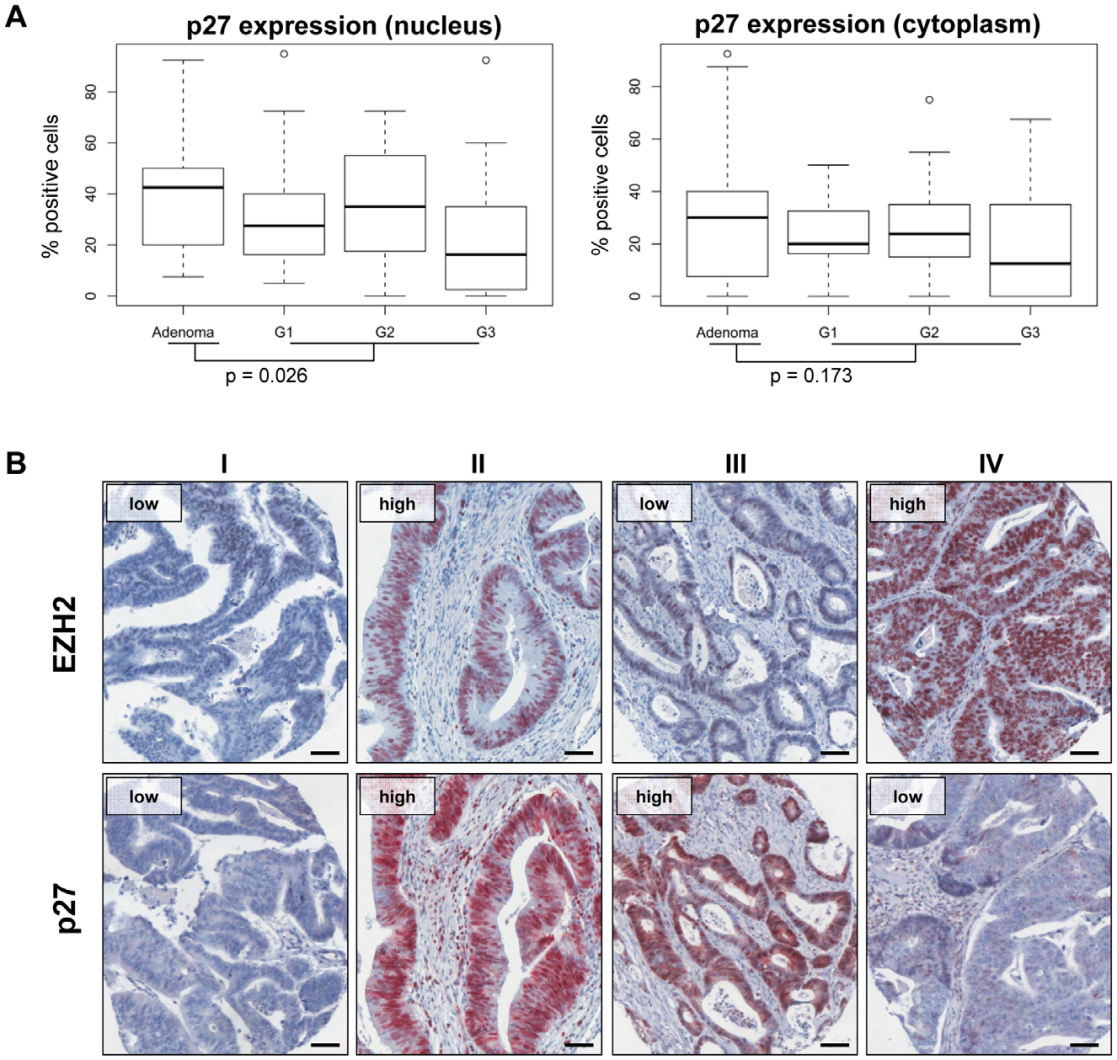
Relation between EZH2 and p27 expression *in vivo*. **A** Box plots of nuclear and cytoplasmatic p27 protein expression in colon adenomas and carcinomas (G1–G3). Expression levels of nuclear p27 were significantly lower in carcinomas than in adenomas (p = 0.026), cytoplasmic p27 levels showed a similar trend, which, however, was not statistically significant (p = 0.173). Differences for p27 expression between G1, G2, and G3 carcinomas were not significant. **B** Immunohistochemical staining of paired samples of colon cancers did not reveal a significant correlation between EZH2 and p27 expression levels. Examples of 4 different cancers (I–IV) stained for EZH2 (upper panels) and p27 (lower panels), respectively. Scale bars, 50 µm.

Immunohistochemical analyses revealed that colon cancers exhibited significantly decreased nuclear p27 and a trend for reduced cytoplasmic p27 protein levels (Figure 5A), when compared with colon adenomas. Within the cancer group, increasing degrees of cancer cell dedifferentiation (G1–G3) showed a statistically non-significant trend for a further decrease of p27 expression (Figure 5A). In general, these results are opposite to the findings obtained for EZH2 expression (Figure 4B), raising the possibility that EZH2 levels may negatively correlate with p27 levels. However, EZH2 and p27 levels did not significantly correlate in individual cancers (n = 68) on a per patient basis, i.e. in tumors derived from the same patient (Figure 5B). This lack of association applied for analyzing EZH2 amounts in relation to both nuclear or cytoplasmic p27 expression levels (Spearman's rank correlation coefficients r = 0.187, p = 0.572 and r = −0.0623, p = 0.613, respectively).

**Figure 6:**
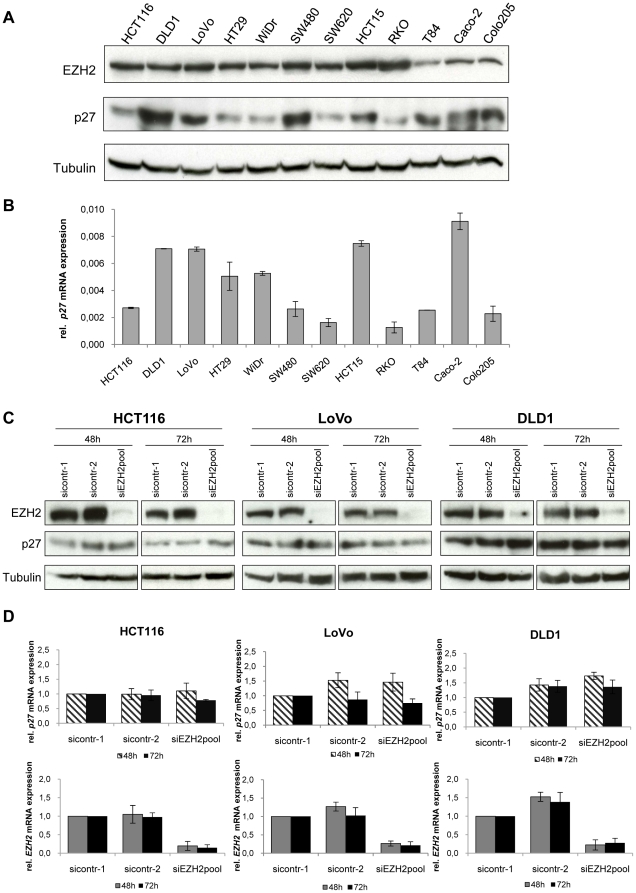
Relation between EZH2 and p27 expression *in vitro*. **A** EZH2 and p27 protein expression in colon cancer cell lines, assessed by immunoblot analyses. Tubulin, loading control. **B** *p27* mRNA expression, measured by qRT-PCR analyses. Data are presented as the fold differences in gene expression, normalized to a housekeeping gene index. Standard deviations from two reverse transcription replicates are indicated. **C** Immunoblot analyses after EZH2 depletion by RNAi. EZH2 and p27 levels are indicated. Tubulin, loading control. **D** qRT-PCR analyses after EZH2 depletion by RNAi. *p27* and *EZH2* mRNA levels are indicated relative to sicontr-1-treated cells (arbitrarily set at 1.0). Standard deviations of three independent experiments are indicated.

In line with these *in vivo* findings, the basal levels of EZH2 protein expression did not consistently correlate with p27 protein or mRNA levels in colon cancer cell lines *in vitro* (Figure 6A and 6B). We also investigated whether p27 expression levels are affected by EZH2 depletion in HCT116, LoVo, and DLD1 colon cancer cells. If EZH2 blocks p27 expression, silencing of *EZH2* should be linked to a re-increase of p27 expression, as has been observed in pancreatic cancer cells [4]. In contrast, however, efficient inhibition of EZH2 expression was not associated with a substantial increase of p27 expression, neither at the protein (Figure 6C) nor at the mRNA (Figure 6D) level, in colon cancer cells. Cellular fractionation studies revealed that p27 is virtually exclusively localized in the cytoplasm, in HCT116, LoVo, and DLD1 cells. This subcellular distribution was also not detectably altered by EZH2 depletion (Figure S1).

### Transcriptome Analyses of Colon Cancer Cells upon EZH2 depletion

**Figure 7:**
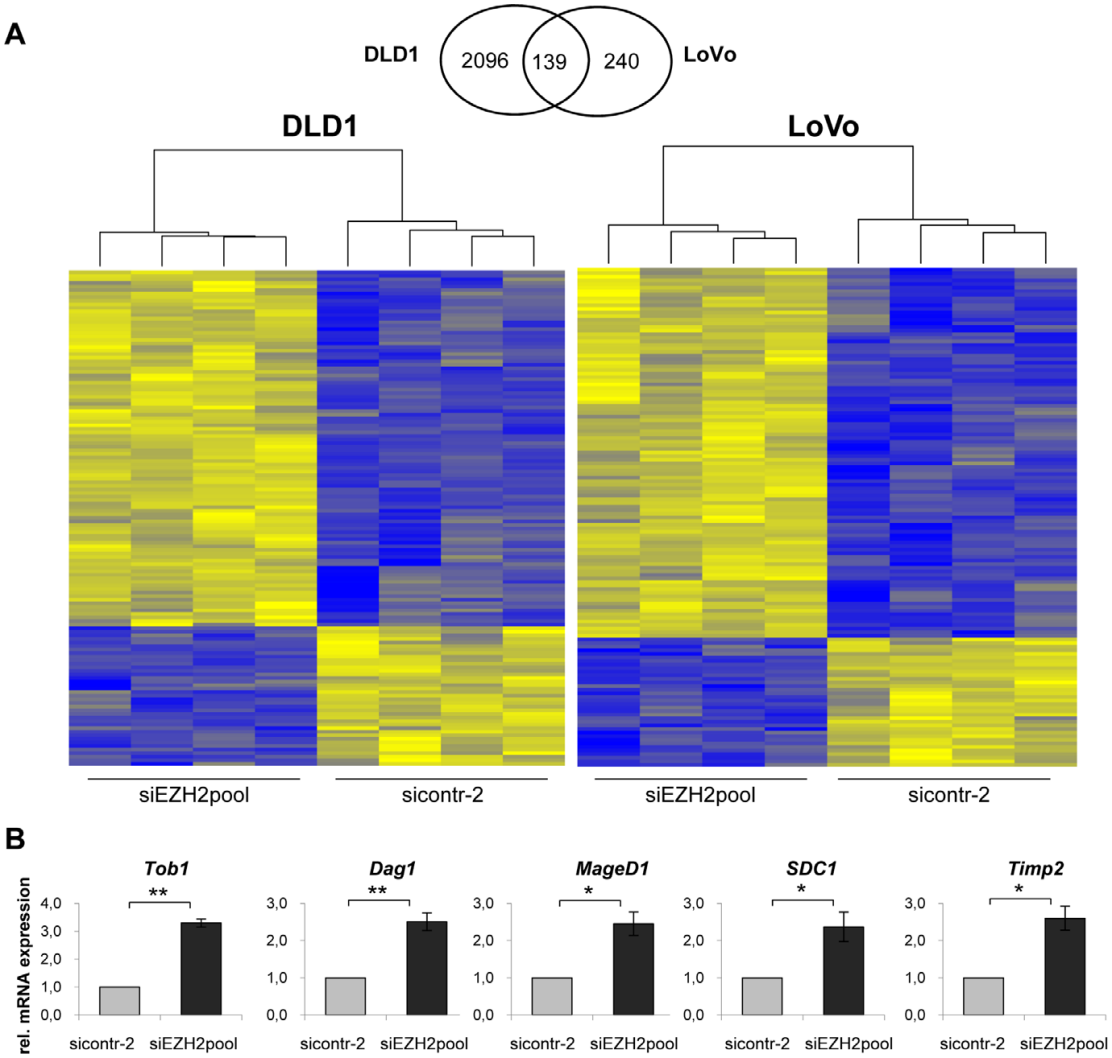
Altered gene expression in colon cancer cell lines upon EZH2 depletion. **A** Venn-Diagram and heatmaps of 139 genes that were significantly affected at the transcript level by EZH2 depletion in both LoVo and DLD1 cells. Heatmaps were generated using hierarchical clustering. Cells were either treated with siEZH2pool or sicontr-2. Four biological replicates were analyzed for each sample. Significantly upregulated genes are indicated in yellow, significantly downregulated genes in blue. **B** qRT-PCR analyses to assess the expression of five genes that were affected by EZH2 depletion in the transcriptome analysis (see above). Indicated are the results from three independent experiments conducted in DLD1 cells. mRNA levels are shown relative to sicontr-2-treated cells (arbitrarily set at 1.0). Standard deviations are indicated. Asterisks equal p≤0.05, double asterisks equal p≤0.01.

In order to identify possible target genes affected by EZH2 depletion in colon cancer cells, transcriptome analyses were performed. To this end, LoVo and DLD1 cells were treated either with the siRNA pool silencing *EZH2* expression or with control siRNA. Changes in the expression levels of cellular genes were assessed by using a genome-wide microarray of approximately 25,000 genes. We observed significant changes of 2,235 genes in DLD1 (1,095 upregulated, 1,140 downregulated) (Table S1) and of 379 genes in LoVo (280 upregulated, 99 downregulated) (Table S2). The overlap consisted of 139 genes that were affected by EZH2 depletion in both colon cancer cell lines (100 upregulated, 39 downregulated). A heatmap visualizing these 139 differentially regulated genes is provided in Figure 7A, indicating high concordance between the biological replicates. A detailed list of these genes is provided in Table S3.

Functional annotation of the 139 genes by Ingenuity Pathway Analysis revealed that 37 gene products have been associated with cancer (Table 1). In regard of the molecular and cellular functions, EZH2 depletion was found to affect several genes involved in the control of cellular development, growth control, cellular movement, and signaling (Table 1).

**Table 1:** IPA analysis of genes affected by EZH2 depletion in both DLD1 and LoVo colon cancer cells.

Diseases and Disorders			
Name	p-Value	# mol.	Genes
Cancer	1.05E-04 - 4.95E-02	37	ANXA5,BCCIP,C15orf48,C8orf84,CMIP,CRAT,DBNL,EGFR,EIF4EBP2,EZH2,FAM57A,FYCO1,FZD7,GNAS,HLA-B,IKBKE,LYAR,MAGED1,MALL,MDK,MFGE8,MRPS30,MYD88,NEU1,NOLC1,NOP16,NPTX2,OLFML3,PLA2G16,PPFIA1,RXRA,SDC1,SRPX,STAT2,TIMP2,TMCO1,TOB1
Infection Mechanism	6.04E-04 - 4.83E-02	12	ANXA5,DAG1,DUSP3,EGFR,EIF4EBP2,IKBKE,MOV10,MYD88,NOLC1,PIK3R2,RXRA,STAT2
Neurological Disease	1.23E-03 - 4.67E-02	8	APLP2,DAG1,EGFR,MYD88,NEU1,SLC25A22,STAT2,TPP1

Molecular and Cellular Functions			
Name	p-Value	# mol.	Genes
Cellular Development	5.19E-04 - 4.69E-02	12	ANXA5,CCDC92,DAG1,EGFR,EZH2,GTPBP4,MAGED1,MYD88,PPFIA1, RXRA,SDC1,TIMP2
Cellular Growth and Proliferation	5.19E-04 - 4.10E-02	12	CCDC92,EGFR,EZH2,FZD7,GTPBP4,MAGED1,MDK,MFGE8,MYD88,RXRA,SDC1,TIMP2
Cellular Movement	9.62E-04 - 4.86E-02	15	APLP2,COL17A1,DBNL,DKK3,EGFR,EZH2,GNAS,MDK,MYD88,NOLC1,PIK3R2, PPFIA1,PPIC,SDC1,TIMP2
Cell-to-cell Signaling and Interaction	2.13E-03 - 4.98E-02	14	ANXA5,COL17A1,DAG1,DKK3,EGFR,EIF4EBP2,GNAS,IQSEC1,MDK,MFGE8,MYD88,RXRA,SDC1,TIMP2

Physiological System Development and Function			
Name	p-Value	# mol.	Genes
Reproductive System Development and Function	5.19E-04 - 3.85E-02	8	ANXA5,CYP26B1,EGFR,EZH2,GNAS,MFGE8,SDC1,TIMP2
Tissue Development	2.28E-03 - 4.26E-02	12	CMIP,DAG1,DKK3,EGFR,GTPBP4,MFGE8,MYD88,RXRA,SDC1,TIMP2,TOB1,TPP1
Connective Tissue Development and Function	3.05E-03 - 4.10E-02	8	EGFR,EIF4EBP2,MDK,MYD88,NPC2,TOB1,TPP1,RXRA
Organ Morphology	3.05E-03 - 3.52E-02	8	EGFR,GNAS,MFGE8,MYD88,RXRA,SDC1,TIMP2,TOB1

mol.: molecules.

To validate the array data, we analyzed the expression of 5 cancer-associated genes, which were induced by EZH2 depletion in the transcriptome analyses, by qRT-PCR: (i) *Dag1* (Dystroglycan 1) encoding an adhesion molecule, which is frequently underexpressed in colon cancer [28], (ii) *MageD1* (Melanoma-associated antigen family protein-D1) encoding an inhibitor of proliferation and tumor cell invasion [29], (iii) *SDC1* (Syndecan 1), encoding a cell surface proteoglycan that inhibits cell invasion [30], (iv) *Timp2* (TIMP metallopeptidase inhibitor 2), encoding an inhibitor of matrix metalloproteinases whose downregulation correlates with the invasive potential of LoVo colon cancer cells [31], and (v) *Tob1* (Transducer of ERBB2), encoding an antiproliferative protein with tumor suppressive potential [32]. As observed for the microarray, all 5 genes were also significantly induced by EZH2 depletion in the qRT-PCR analysis (Figure 7B), further corroborating the transcriptome data.

## Discussion

In the present study, we show that EZH2 depletion in colon cancer cells (i) reduces cell cycle progression at the G1/S boundary, (ii) decreases cell numbers in short term growth assays, and (iii) blocks cell growth in long-term colony formation assays. These results are consistent with a growth-promoting role for EZH2 in colon cancer and are in contrast to a recent report indicating that the growth of colon cancer cells is not affected by siRNA-mediated EZH2 depletion [22].

A possible explanation for this discrepancy may be related to the assays used to measure cell growth. The previous study relied on the MTT assay that measures a metabolic activity (reduction of MTT (3-(4,5-Dimethylthiazol-2-yl)-2,5-diphenyltetrazolium bromide to formazan) [22]. This assay can be affected by many conditions, e.g. metabolic changes, and can lead to the underestimation of growth inhibitory effects [33]–[35]. In contrast, in the present study, antiproliferative effects following *EZH2* silencing were consistently observed in three independent assays. Our findings thus indicate that EZH2 stimulates the proliferation of colon cancer cells, as has been reported for several other cancer entities [2]–[9], [11].

The exact molecular mechanisms how EZH2 stimulates cell proliferation are still largely unknown. As a crucial component of the PRC2 transcriptional repressor complex, EZH2 may lead to the repression of antiproliferative genes. An interesting study recently demonstrated that EZH2 leads to the repression of the growth-inhibitory *p27* cell cycle regulator gene in pancreatic cancer cells [4]. Since p27 acts at the G1/S transition - which we found to be affected by *EZH2* silencing in colon cancer cells - and since p27 levels are frequently low in colon cancers [26], [27], we tested a possible correlation between EZH2 and p27 levels *in vivo* and *in vitro*.

However, comparative analyses of EZH2 and p27 expression did not exhibit a statistically relevant positive or negative linkage in colon cancers *in vivo*, on a per patient basis. Moreover, EZH2 depletion did not result in a re-expression of p27 in colon cancer cell lines *in vitro*, at time points where it resulted in highly increased p27 expression in pancreatic cancer cell lines [4]. These findings indicate that - in contrast to the situation in pancreatic cancer cells - p27 levels are not critically regulated by EZH2 in colon cancer cells. Thus, the spectrum of EZH2 target genes may vary in a tissue- or cell-specific manner.

In order to get insight into the spectrum of genes, which are affected by EZH2 depletion in colon cancer cells, we performed whole genome transcriptome analyses in DLD1 and LoVo cells. Among the 139 genes, which were significantly affected in both cell lines, over one fourth is known to be cancer-associated. The spectrum of affected genes is consistent with the hypothesis that EZH2 is an important factor for development, proliferation control, signaling, and movement/invasion [1], [36]. Additional work is required to analyze the exact mechanisms by which EZH2 may alter the expression of these genes and to study in detail their possible contribution to the growth deregulation of colon cancer cells. We corroborated the array data by expression analysis of 5 genes by qRT-PCR: *Dag1*, *MageD1*, *SDC1*, *Timp2*, and *Tob1*. In line with the transcriptome analysis, *EZH2* silencing increased the expression of all 5 genes in qRT-PCR analyses as well. These findings suggest that EZH2 may repress these genes either directly or indirectly, in colon cancer cells. All 5 genes are reported to exhibit antiproliferative and/or antiinvasive potential [28]–[32] and their downregulation would thus be consistent with a possible oncogenic effect of EZH2 in colon cancer.

Due to the growth-promoting role of EZH2 in various cancers, inhibition of EZH2 is currently discussed as an attractive novel strategy for cancer therapy [1], [12]. Indeed, *EZH2* inhibition by siRNAs, or depletion of PRC2 components by the drug 3-deazaneplanocin A (DZNep), exerted antioncogenic effects, by blocking cell proliferation and/or inducing apoptosis [2]–[7], [11], [37], [38], by counteracting invasion and metastasis [9], [10], and by inhibiting tumor angiogenesis [8].

The finding in our study that EZH2 contributes to the proliferation of colon cancer cells extends the spectrum of tumor entities that may therapeutically benefit from EZH2 inhibition to colon cancer. Considering EZH2 as a potential therapeutic target, however, must take into account that EZH2 is also expressed in normal tissues, including the proliferative cell layer of colon crypts [22]. Importantly, EZH2 has been found to exert crucial regulatory functions in several tissues, such as controlling the differentiation of tissue-specific progenitor and stem cells [39]–[42]. Moreover, the recent observation that EZH2 may act as a tumor suppressor in certain hematologic disorders [14], [15], [16] suggests that EZH2 inhibition could even promote tumorigenesis, in some tissues. Thus, interfering with EZH2 function as a therapeutic strategy bears the risk to induce unwanted side effects and is likely to require highly specific delivery of EZH2 inhibitors to their target cells.

## Materials and Methods

### Cells and transfections

HCT116, DLD1, LoVo, WiDr, SW620, HCT15, RKO, T84, Caco-2, and COLO205 colon carcinoma cell lines were a kind gift from Dr. M. von Knebel Doeberitz (University of Heidelberg), SW480 from Dr. S. Wiemann (German Cancer Research Center, Heidelberg), and HT29 from the cell and tissue culture core facility of the German Cancer Research Center, Heidelberg. Cells were maintained in either DMEM (pH 7.2), RPMI, or McCoys medium, supplemented with 10% FCS, 50 units/ml penicillin, and 50 µg/ml streptomycin sulfate.

Plasmids were transfected by calcium phosphate coprecipitation [43] into HCT116 cells or by Fugene HD (Roche Diagnostics, Mannheim, Germany) into DLD1, LoVo, and RKO cells. Synthetic siRNAs were transfected with Dharmafect (Dharmacon, Thermo Fisher Scientific, Lafayette, CO, USA) according to the manufacturer's protocol. In brief, cells were plated in 6-cm dishes at 15% to 25% confluency. Dharmafect 4 and siRNAs (final concentration of 100 nM) were both diluted in Opti-MEM I reduced serum medium (Invitrogen, Carlsbad, CA, USA) and mixed in a volume of 400 µl transfection solution.

### Plasmids and synthetic siRNAs

siRNAs were either chemically synthesized (Dharmacon) or expressed as shRNAs from pCEPsh, as previously described [44]. The following *EZH2*-targeting siRNAs were used: siEZH2-1 5′-GAAUGGAAACAGCGAAGGA-3′ (predesigned siRNA from Dharmacon), siEZH2-2 5′-GACUCUGAAUGCAGUUGCU-3′ [7], and siEZH2-3 5′-GCUGAAGCCUCAAUGUUUA-3′ (predesigned siRNA from Dharmacon). The siEZH2pool consisted of all three siRNAs mixed at equimolar concentrations. The following control siRNAs were used: sicontr-1 5′-CAGUCGCGUUUGCGACUGG-3′ [45], sicontr-2 5′-UAGCGACUAAACACAUCAA-3′ (predesigned siRNA from Dharmacon, containing at least four mismatches to all known human genes), and siluc 5′-CAUCACGUACGCGGAAUAC-3′ (targeting *Photinus pyralis* luciferase [46]).

### RNA extraction, quantitative real-time reverse transcription polymerase chain reaction (qRT-PCR), and protein analyses

RNA was isolated as previously described [47] and resuspended in RNAse free water. RNA concentrations were measured with NanoDrop ND-1000 (Thermo Fisher Scientific, Wilmington, DE USA), at 260 nm. Reverse transcription of 1 µg RNA was performed by using the oligo-dT primer and ProtoScript M-MuLV Taq RT-PCR Kit (New England Biolabs, Frankfurt, Germany) according to the manufacturer's protocol. Expression levels were determined by real-time PCR with a 7300 Real-Time PCR System detector (Applied Biosystems, Carlsbad, CA, USA), using the SYBR green PCR Master Mix (Applied Biosystems), supplemented to 500 nM of each forward and reverse primer. *EZH2* (NM_004456) expression was determined using the forward primer 5′-TTGTTGGCGGAAGCGTGTAAAATC-3′ and reverse primer 5′-TCCCTAGTCCCGCGCAATGAGC-3′ [48]. For detection of *p27^Kip1^* (NM_004064) expression 5′-GCCAGACGGGGTTAGCGGAG-3′ was used as forward and 5′-GAGGCCAGGCTTCTTGGGCG-3′ as reverse primer. *MageD1* (NM_001005333) expression was determined with primers 5′-GGCTGTCCTCTGGGAGGCACT-3′ and 5′-GGGTTGCTGTTGGGCACTCGT-3′, *Timp2* (NM_003255) expression with primers 5′-TCTACACGGCCCCCTCCTCG-3′ and 5′-TGGGGCAGCGCGTGATCTTG-3′, *SDC1* (NM_001006946) expression with primers 5′-CGGCCCTGCCGCAAATTGTG-3′ and 5′-CCTCCAGGCCGGTGGGTTCT-3′, *Tob1* (NM_005749) expression with primers 5′-TGCAGCCTATGGAGGCCTCAA-3′ and 5′-CCCCTTGGGCCCGTGCATTTT-3′, and *Dag1* (NM_001165928) expression with primers 5′-GTCGTCGGGCGCTCATTTCGA-3′ and 5′-CCAGCCGTGTAGCGCTCACTG-3′. GAPDH and HPRT1 primer sequences and cycling conditions have been previously described [49]. The sizes of the PCR products were initially verified by agarose gel electrophoresis and subsequently checked by melting point analysis after each reaction. Relative quantification was performed using the comparative Ct (2^−ΔΔCt^) method [50]. Data are presented as the fold difference in gene expression normalized to a housekeeping gene index (the geometric mean of *GAPDH* and *HPRT1* expression levels), and relative to a calibrator sample. The housekeeping genes were chosen among several tested housekeeping genes for normalization of gene expression, since they exhibited equal amplification efficiencies as our genes of interest. Statistical significance of differences in measured variables between controls and treated groups was determined by a two-sided paired t-test using the Sigma Plot software (Systat Software Inc., San Jose, CA). Differences were considered significant at p≤0.05.

Total protein extracts were prepared 48 to 96 hours after transfection, as described previously [51]. For cytosolic and nuclear extract preparation, cells were resuspended in lysis buffer (10 mM Tris, 10 mM NaCl, 1 mM EDTA, 0.5% Nonidet® P-40, pH 7.4) and incubated on ice. Intact nuclei were pelleted by centrifugation and the cytosolic extract in the supernatant was transferred. Nuclei were washed twice with lysis buffer containing 0.05% Nonidet® P-40 and the nuclear proteins were extracted as described for total protein extracts. For Western blot analyses, 20–30 µg of protein extract were separated by 12.5% SDS-PAGE, transferred to an Immobilon-P membrane (Millipore, Bedford, MA, USA), and analyzed by enhanced chemiluminescence (GE Healthcare, Buckinghamshire, UK). The following antibodies were used: anti-EZH2 antibody (AC22, Cell Signaling, Danvers, MA, USA), anti-p27 antibody (#610242, BD Transduction Laboratories, Franklin Lakes, NJ, USA), anti-cyclin D1 antibody (DSC6, Cell Signaling), and anti-alpha-tubulin antibody (CP06, Calbiochem, Darmstadt, Germany).

### Cell count and cell cycle analyses

For cell count analyses, total cell numbers were determined 48–72 hours after transfection. Total cells per milliliter were measured, using a Countess Cell Counter (Invitrogen).

For cell cycle analyses, cells were trypsinized 48 hours after transfection, washed in ice-cold phosphate-buffered saline (PBS), and fixed in 80% cold ethanol overnight at −20°C. Subsequently, cells were pelleted, resuspended in PBS containing 1 mg/ml RNAse A (Roche Diagnostics) and 25 µg/ml propidium iodide (Sigma-Aldrich, Munich, Germany), and then incubated for 30 min at 37°C. Cell cycle analyses were performed using a FACSCalibur (BD Biosciences, Heidelberg, Germany) with CellQuest Pro software provided by the manufacturer. Apoptotic cells were excluded and quantitation of the percentage of cells in individual cell cycle phases was performed using FlowJo software (Tree Star, Ashland, OR), applying the Dean-Jett-Fox model [52]. Statistical significance of differences in measured variables between controls and treated groups was determined by a two-sided paired t-test as described above.

### Colony formation assay

For colony formation assays, cells were grown on 6 cm dishes and transfected with individual pCEPsh vectors. Colonies were fixed and stained with formaldehyde-crystal violet 13–15 days after transfection and subsequent selection for hygromycin B (Sigma) resistance.

### Tissue Micro Array

A tissue microarray (TMA) containing tissue samples derived from tubular adenomas (n = 30) and G1 (n = 30), G2 (n = 30), and G3 (n = 30) colorectal adenocarcinomas was analyzed. All tissue samples were obtained from the Tissue Bank of the National Center for Tumor Diseases (NCT) Heidelberg after approval by the ethics committee of the University of Heidelberg.

For the creation of the TMA, representative tissue blocks were selected as donor blocks. Sections were cut from each donor block and stained with Hematoxylin and Eosin. Then, a morphologically representative region was chosen from each tissue sample. One cylindrical core tissue specimen per block (diameter 0.6 mm) was punched from these regions and arrayed into the recipient paraffin block using a semiautomatic system Tissue Arrayer MTA-1 (AlphaMetrix, Rödermarkt, Germany).

### Immunohistochemistry

The TMA slides were dewaxed and rehydrated using xylene and a series of graded alcohols, followed by heat induced antigen retrieval using a target retrieval solution (S2031, DakoCytomation, Glostrup, Denmark) in a pressure cooker for 10 min. Immunohistochemical staining was performed on an automated staining system (Techmate 500, DakoCytomation) with a mouse anti-EZH2 antibody (1∶20, 612667, BD Transduction Laboratories) for 30 min, and an mouse anti-p27 antibody (1∶150, 610242, BD Transduction Laboratories) for 30 min. An avidin-biotin-complex peroxidase technique using aminoethylcarbazole for visualisation and Hematoxylin for counterstaining was applied. According to the manufacturers' instructions, the following solutions were used: ChemMate Detection Kit (K5003, DakoCytomation), ChemMate Buffer Kit (K5006, DakoCytomation) and, for reduction of non-specific avidin/biotin-related staining, the Avidin/Biotin Blocking Kit (SP-2001, Vector Laboratories, Burlingame, USA). Reactive infiltrating lymphocytes, which express detectable amounts of EZH2 protein, served as a internal positive control [53]. As a negative control for the immunohistochemical staining procedure, the primary antibody was omitted, with all other experimental conditions kept constant.

For immunohistochemical measurement of EZH2 expression, the frequency of nuclear staining was evaluated. p27 staining was determined separately for nuclear and cytoplasmic expression. Due to insufficient tumour tissue or fixation artefacts, which interfered with immunohistochemical staining, 23 cases for EZH2-staining and 18 cases for p27-staining were excluded from further analyses. The arrays were independently scored by two researchers, blinded for tissue annotation. For the few instances of discrepant scoring, a consensus score was determined. Box plots were drawn as described in [54]. The association of p27 and EZH2 expression and histological grading (Adenoma, G1, G2, G3) was analyzed using a two-stage approach. First the two-sided Jonckheere-Terpstra test was applied. If a statistically significant trend was found (i) the Mann-Whitney test was used to compare between adenomas and carcinomas and (ii) the Jonckheere-Terpstra test was applied to the subgroup of carcinomas to test for a trend according to histological grading. To measure the correlation of p27 and EZH2 expression in colon carcinomas Spearman's rank correlation coefficient was calculated. A result was considered as statistically significant, if the p value was smaller than or equal to 5%. All statistical analyses were performed within the R statistical software environment (R version 2.11.1) using the R package coin, version 1.0-11.

### Transcriptome analyses

For gene expression analysis, we used the whole genome expression microarray Sentrix® HumanHT-12 v4 expression bead chip (Illumina®, San Diego, CA, USA) encompassing 47,231 features. The experiments were performed at the Genomics and Proteomics Core Facility of the German Cancer Research Center, Heidelberg, using concentrations of 50 ng/µl RNA for each sample and following the protocols recommended by the supplier. Expression levels were analyzed 48 hours post transfection for DLD1 cells and 72 hours post transfection for LoVo cells.

Statistical analysis was performed using the statistical computing environment R [55]. Briefly, the gene expression profiles were normalized using quantile normalization and differentially expressed genes were determined using a moderated t-statistic [56]. All p-values were corrected for multiple testing, and genes showing a false discovery rate [57] p≤0.05 were considered as significantly deregulated. The statistical analysis was performed for each cell line separately and for further analysis only the genes that were significantly affected in both cell lines were used. The functional analyses were generated through the use of IPA (Ingenuity Systems, www.ingenuity.com).

## Supporting Information

Figure S1 **Subcellular localization of p27 in colon cancer cells.** Immunoblot analysis of p27 and EZH2 in cytoplasmic and nuclear extracts prepared from colon cancer cells. Cells were harvested 48 hours following treatment with control siRNAs (sicontr-1 and sicontr-2) or siEZH2 pool. Fractionation controls: Lamin A/C (nuclear protein) and Tubulin (cytoplasmic protein).
(TIF)

Table S1 **Transcripts significantly affected upon EZH2 depletion in DLD1 cells.**
(XLS)

Table S2 **Transcripts significantly affected upon EZH2 depletion in LoVo cells.**
(XLS)

Table S3 **Transcripts significantly affected upon EZH2 depletion in both LoVo and DLD1 cells.**
(XLS)
