# Supplementary figures and images for: EZH2 Depletion Blocks the Proliferation of Colon Cancer Cells
Source: PLoS One. 2011 Jul 13;6(7):e21651. doi: 10.1371/journal.pone.0021651 (PMC3135584; doi:10.1371/journal.pone.0021651)

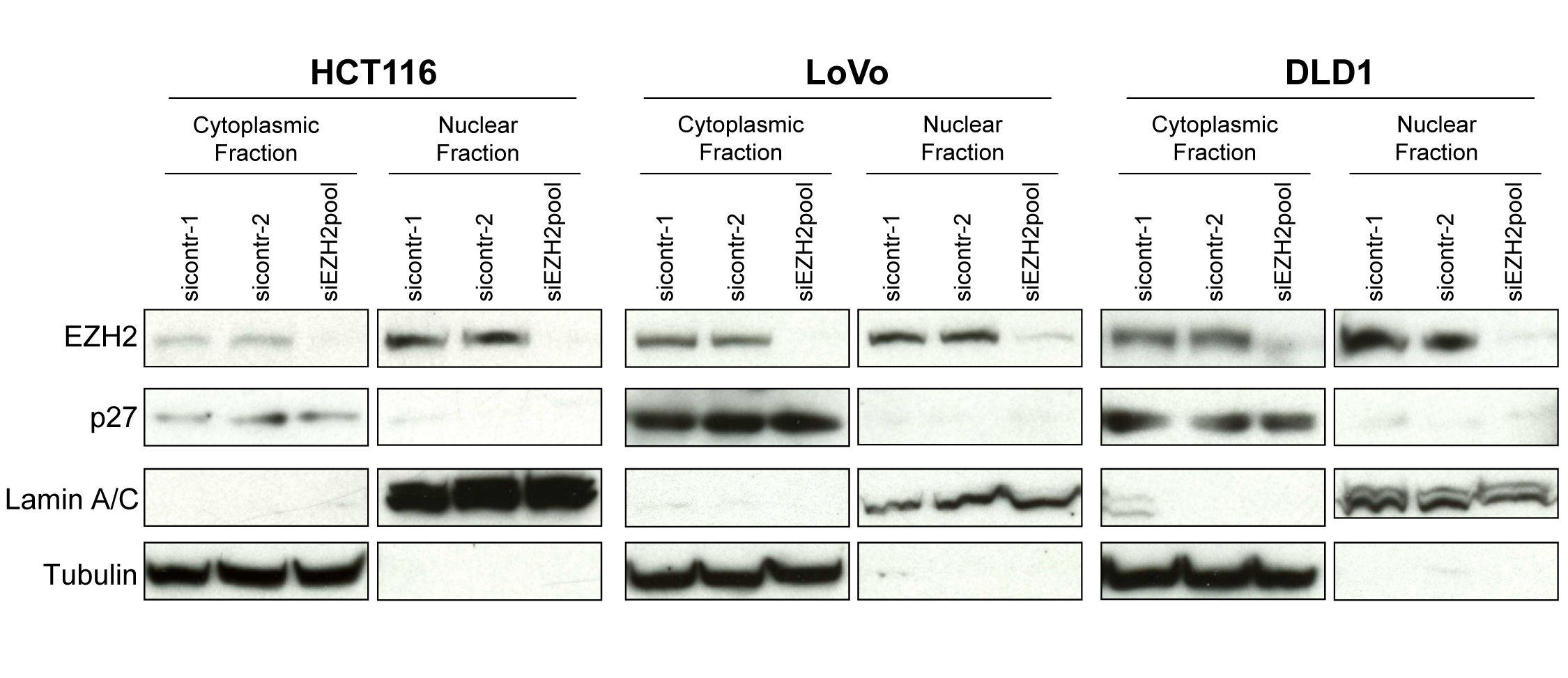

Supplement: Figure S1 — Subcellular localization of p27 in colon cancer cells. Immunoblot analysis of p27 and EZH2 in cytoplasmic and nuclear extracts prepared from colon cancer cells. Cells were harvested 48 hours following treatment with control siRNAs (sicontr-1 and sicontr-2) or siEZH2 pool. Fractionation controls: Lamin A/C (nuclear protein) and Tubulin (cytoplasmic protein). (TIF) [file pone.0021651.s001.tif]
